# Plant F-Box Protein Evolution Is Determined by Lineage-Specific Timing of Major Gene Family Expansion Waves

**DOI:** 10.1371/journal.pone.0068672

**Published:** 2013-07-19

**Authors:** Aura Navarro-Quezada, Nadine Schumann, Marcel Quint

**Affiliations:** 1 Department of Stress and Developmental Biology, Leibniz Institute of Plant Biochemistry, Halle (Saale), Germany; 2 Department of Molecular Signal Processing, Leibniz Institute of Plant Biochemistry, Halle (Saale), Germany; 3 Department of Molecular Ecology, Max Planck Institute for Chemical Ecology, Jena, Germany; University of Nottingham, United Kingdom

## Abstract

F-box proteins (FBPs) represent one of the largest and fastest evolving gene/protein families in the plant kingdom. The FBP superfamily can be divided in several subfamilies characterized by different C-terminal protein-protein interaction domains that recruit targets for proteasomal degradation. Hence, a clear picture of their phylogeny and molecular evolution is of special interest for the general understanding of evolutionary histories of multi-domain and/or large protein families in plants. In an effort to further understand the molecular evolution of F-box family proteins, we asked whether the largest subfamily in *Arabidopsis thaliana*, which carries a C-terminal F-box associated domain (FBA proteins) shares evolutionary patterns and signatures of selection with other FBPs. To address this question, we applied phylogenetic and molecular evolution analyses in combination with the evaluation of transcriptional profiles. Based on the 2219 FBA proteins we *de novo* identified in 34 completely sequenced plant genomes, we compared their evolutionary patterns to a previously analyzed large subfamily carrying C-terminal kelch repeats. We found that these two large FBP subfamilies generally tend to evolve by massive waves of duplication, followed by sequence conservation of the F-box domain and sequence diversification of the target recruiting domain. We conclude that the earlier in evolutionary time a major wave of expansion occurred, the more pronounced these selection signatures are. As a consequence, when performing cross species comparisons among FBP subfamilies, significant differences will be observed in the selective signatures of protein-protein interaction domains. Depending on the species, the investigated subfamilies comprise up to 45% of the complete superfamily, indicating that other subfamilies possibly follow similar modes of evolution.

## Introduction

An important post-translational regulatory mechanism is protein degradation. In eukaryotes, a major player in this process is the ubiquitin-proteasome system. In addition to the removal of misfolded proteins, it allows living organisms to adapt to changing environments by providing fast responses to intracellular signals. The largest class of ubiquitin ligases in plants are Skp1-Cullin-F-box protein (SCF) complexes. F-box proteins (FBPs) are the target-recruiting subunits of SCF complexes and confer selectivity to the whole system [1]. Compared to most other eukaryotic model organisms, the FBP superfamily expanded dramatically in land plants, making it an attractive subject for studying mechanisms that shape the molecular evolution of gene families. With on average several hundred members in each species [2], FBPs represent one of the largest and fastest evolving protein families in the plant kingdom [3]. The FBP superfamily has previously been studied on an evolutionary scale and comprises several subfamilies that differ in their C-terminal protein-protein interaction domain [4]–[6]. However, simultaneous analysis of sometimes >1000 FBPs per species with a variety of different C-terminal domains can only provide a glimpse on the characteristics of the various subfamilies. Hence, little is known about the molecular evolution of specific subfamilies of FBPs. The three largest FBP subfamilies in the model plant *A. thaliana* carry either F-box associated domains (Pfam domains PF04300 and PF08268, from here on referred to as FBA-D), leucine-rich repeats, or kelch repeats C-terminal to the F-box. As these different subfamilies probably emerged very early during the evolution of the FBP superfamily, they might have evolved very differently. We previously analyzed molecular evolution and selection patterns of FBPs with additional kelch repeat domains (FBK proteins). Although present throughout eukaryotes, the FBK protein subfamily expanded dramatically in land plants with various selective signatures depending on the evolutionary conservation of the respective phylogenetic clade. Furthermore, F-box and kelch domains seem to be under opposite selective pressures [7].

The largest FBP subfamily with >200 members in *A. thaliana* carries a C-terminal FBA-D [2], [6]. So far, six *A. thaliana* FBPs with FBA-Ds (from here on referred to as FBA proteins or genes) have been functionally characterized and are involved in pathogen responses [8], [9] root development [10], ethylene signaling [11], and abscisic acid responses [12]. While nothing is known about the structural features of this domain, sequence similarity to kelch repeat motifs indicates a function as protein-protein interaction domain, possibly by forming similar propeller-like structures [7].

To identify common evolutionary patterns as well as possible differences between FBP subfamilies, we analyzed the molecular evolution and selection patterns acting on *FBA* genes and compared the results to what we had previously learned from the *FBK* gene subfamily. These two subfamilies account for ∼45% of the FBPs in the model species *A. thaliana.* We thus assume that comparison of these subfamilies can aid in making general inferences about F-box gene family evolution. We first screened the genomes of 34 green algae and land plant species, as well as other eukaryotic model organisms for the presence of FBA proteins by employing a Hidden Markov Model-based search. We then reconstructed the phylogeny of the same seven land plant species and a unicellular alga we previously analyzed for FBK protein evolution [7], and analyzed patterns of selection acting on the F-box domain and the FBA-D. These analyses were complemented by investigating spatio-temporal expression profiles of the *FBA* gene paralogs from *A. thaliana* across different developmental stages and various stress responses.

## Results

### FBA proteins are present in all investigated land plant species and one unicellular alga

**Figure 1:**
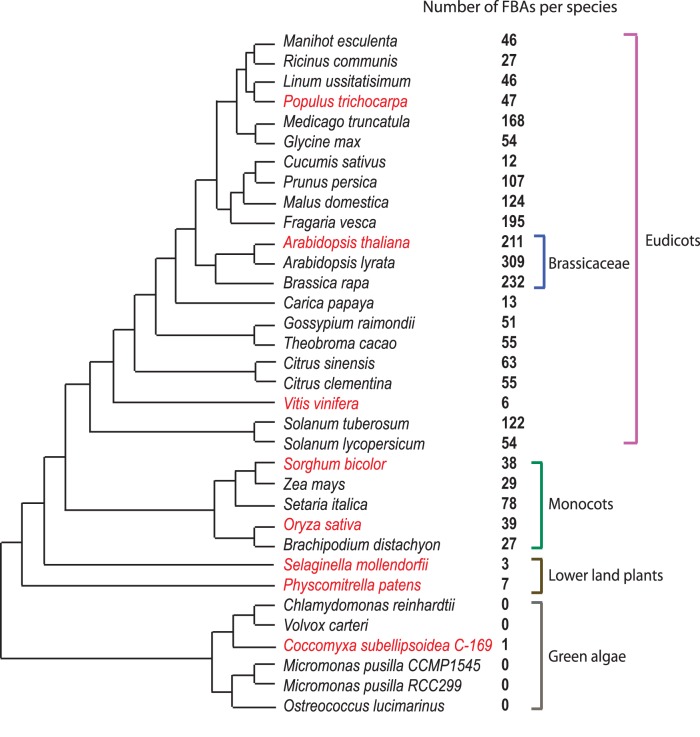
Phylogeny of species included in this analysis and number of FBA proteins identified. The tree represents the phylogenetic relationships between the species analyzed. Branch lengths are not in proportion to evolutionary time. The number of FBA proteins identified per species is indicated next to the species name. Species included in the phylogenetic reconstruction of FBA protein evolution are indicated.

The Hidden Markov Model search allowed us to identify a total of 2219 FBA proteins in the genomes of 28 land plant species and six green algae (Figure 1). At the base of the green plant lineage most algal genomes do not contain FBA proteins at all. The only exception seems to be the uni-cellular alga *Coccomyxa* sp. C-169 with a single FBA protein (Fig. 1). In contrast, the FBA protein family apparently expanded significantly during or after land colonization. The so-called ‘lower’ land plant species that we include in our screen, the bryophyte *Physcomitrella patens* and the lycophyte *Selaginella moellendorffii*, contain seven and three FBA proteins, respectively (Fig. 1). In higher plants, we noticed a more extensive expansion in the number of FBA genes/proteins. Here, we identified between 27 and 78 FBA proteins in the five investigated monocot genomes. Gene family expansion varies dramatically in the 21 eudicot genomes ranging from very limited expansion in *Vitis vinifera* (six FBA genes/proteins) to extensive expansion in *Arabidopsis lyrata* with 309 gene family members (Fig. 1). Given the contrasting and increasing numbers of FBA genes/proteins along plant evolutionary history, lineage-specific expansion seems to be an important characteristic of this particular FBP subfamily, as can be seen especially for the Brassicaceaes (Fig. 1).

### The phylogenetic tree identifies FBAs with different evolutionary stabilities

**Figure 2:**
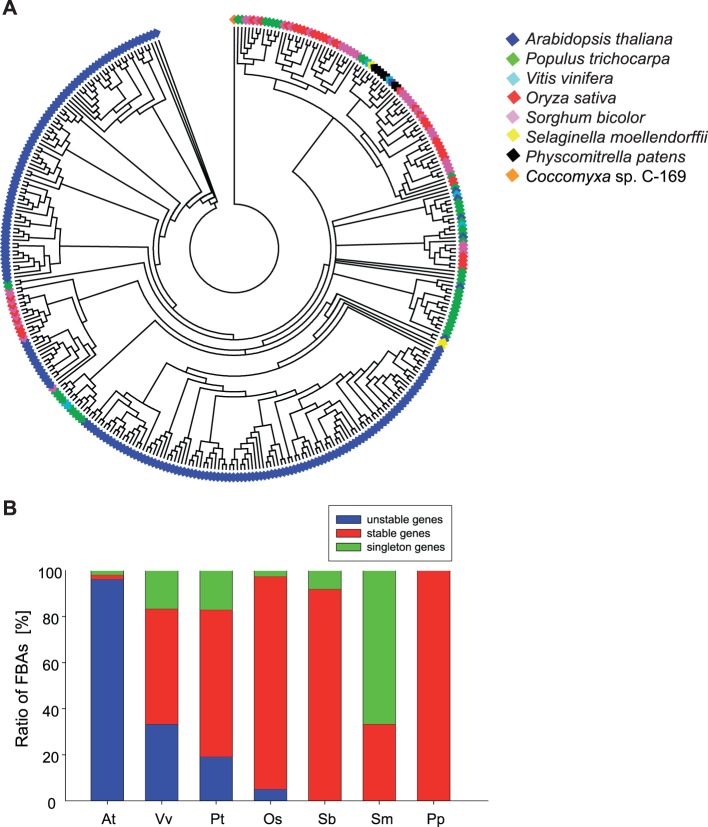
Phylogeny of F-box proteins with C-terminal FBA domains in land plant species. A, Phylogenetic tree of *A. thaliana, V. vinifera, P. trichocarpa, O. sativa, S. bicolor, S. moellendorffii* and *P. patens* FBA proteins. Multiple sequence alignments of the full-length FBA protein sequences were performed using MUSCLE. The phylogenetic tree was generated using ML methods in GARLI. The tree was rooted with the FBA protein sequence of *Coccomyxa* sp. C-169. The color code corresponds to the different species. B, Ratios of unstable, stable and singleton FBA proteins in the seven analyzed land plant species.

If lineage-specific expansion determined the evolutionary fate of the *FBA* genes, we expected to estimate the time of the *FBA* gene duplication events by reconstructing a robust phylogeny using maximum likelihood (ML) methods implemented in GARLI (Fig. 2a, [13]). To allow for optimum comparison to the phylogenetic patterns identified for the FBK protein subfamily, we generated the FBA protein phylogeny for exactly the same seven land plant species as previously investigated for FBK proteins (*A. thaliana*, *V. vinifera*, *P. trichocarpa*, *O. sativa*, *S. bicolor*, *S. moellendorffii*, *P. patens*) and one algal species. In total, these species contained 352 FBA proteins. The resulting phylogenetic tree was generated on basis of full-length protein sequences and rooted with the FBA protein sequence of the green alga *Coccomyxa* sp. C-169 (a subsample of the alignment is shown in Fig. S1). Hence, it reflects the evolution of both the F-box domain and the FBA-D. To test the validity of the tree topology, we next generated phylogenetic trees with randomly chosen representative FBA proteins based on ML, Bayesian inference, and Neighbor-joining (NJ) methods (Fig. S2). All three topologies are very similar and not significantly different, and therefore support the topology of the large tree (Fig. 2a, Table S2 and Fig. S3).

Hence, by revealing the phylogenetic architecture of the FBA protein subfamily between and within species the ML tree contains valuable information on several levels. Generally, FBA proteins from the same species tend to cluster together. However, the phylogeny also contains clades that are conserved to various degrees across species. In total, we observe 26 well supported clades, which we classified into categories that reflect the mode of evolution of the FBA proteins therein (Table 1 and Fig. S3). Clades with proteins from several species are hypothesized to display significant functional conservation [14], possibly by regulating ancient pathways in plant development or physiology. The highest degree of evolutionary conservation would be represented by clades containing FBA proteins from all seven analyzed land plant species, indicating that loss of such a gene/protein would mean a fitness disadvantage for all tested land plant species. In contrast to our previous study on FBK proteins [7], we did not identify such evolutionary ‘superstable’ clades in the FBA protein phylogeny.

**Table 1:** Number of unstable, stable and singleton FBA proteins in *A. thaliana*, *V. vinifera*, *P. trichocarpa*, *O. sativa*, *S. bicolor*, *S. moellendorffii*, *P. patens, and C.* sp. C-169.

Species	Unstable	Stable	Singleton	Total
*Arabidopsis thaliana*	203	4	4	211
*Vitis vinifera*	2	3	1	6
*Populus trichocarpa*	9	30	8	47
*Oryza sativa*	2	36	1	39
*Sorghum bicolor*	0	34	3	37
*Selaginella moellendorffii*	0	1	2	3
*Physcomitrella patens*	0	7	0	7
*Coccomyxa* sp. C-169	0	0	1	1

Clades containing proteins of more than one species, are designated evolutionary ‘stable’, suggesting an important role in a lineage comprising several species. In total, roughly one third of the FBA proteins (115 of 352) fall into the stable category (Table 1). The remaining 67% (238 of 352) are species-specific, either paralogs or singletons, without clear orthologs in any other analyzed species. According to Schumann et al. [7] proteins in this category are considered evolutionary ‘unstable’.

A comparison of the different species analyzed in this study shows that the distribution of the FBA proteins in the stable/unstable categories varies greatly. Unstable *FBA* genes are dominating the *A. thaliana* genome (96%, Fig. 1b), indicative of recent expansion. In contrast to this, the genomes of the lower land plants completely lack unstable genes, suggesting that FBA genes/proteins retained in these species have rather conserved functions. However, we are aware that the identification of unstable genes might be affected by the set of species included in the analysis. Therefore, these data have to be carefully interpreted for the two Poaceaes *O. sativa* and *S. bicolor*, because they are more closely related than, for example, *A. thaliana* and *P. trichocarpa* (the two grasses diverged 40 mya, the two eudicots diverged 100 mya; timetree.org), Hence, the existence of unstable genes as we defined them is less likely for species with close relatives among the species analyzed. In contrast to the grasses, the two lower land plant species (which also lack unstable FBA genes/proteins) should be resistant to this potential bias, because they are not closely related to any of the other species analyzed.

### *FBA* gene expansion occurred mostly in angiosperms

**Figure 3:**
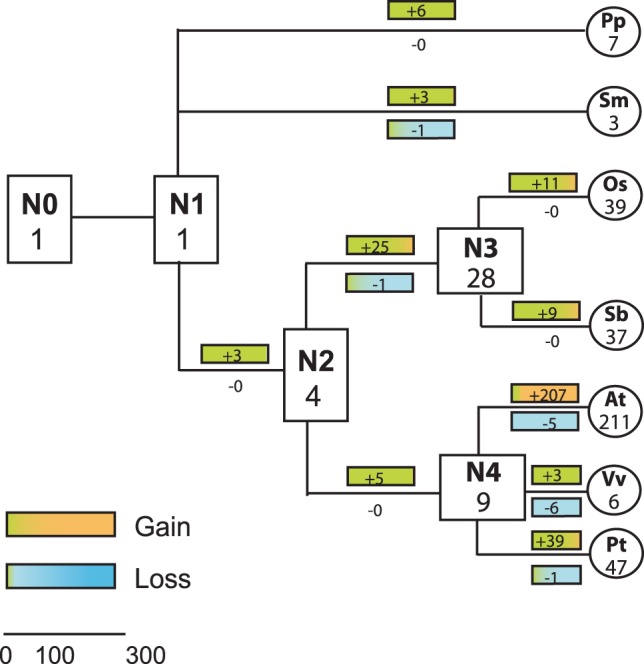
Evolutionary change in the number of FBA proteins in land plants. The numbers in rectangles and circles represent the maximum number of genes in ancestral and extant species, respectively. The numbers with plus and minus signs indicate gene gains and losses, respectively, for each branch. Bold lines represent branches with high gene expansion rate. N0: Chloroplastida ancestor, N1: land plant ancestor, N2: angiosperm ancestor, N3: monocot ancestor, N4: eudicot ancestor. Branch lengths are not in proportion to evolutionary time.

The phylogenetic analysis based on the FBA protein sequences allows reconstruction of the duplication events of the *FBA* genes in the different lineages over evolutionary time. Particularly, we estimated the number of *FBA* genes/proteins in the most recent common ancestor (MRCA) of the seven analyzed land plant species and determined the number of gained and lost genes. The 26 clades identified from the phylogenetic tree contain mostly orthologous genes (Fig. S3), most of which are not present in the potential MRCAs of the seven analyzed species (Fig. 3). At the base of the tree we find the green alga *Coccomyxa* sp. C-169, which, as mentioned above, is the only sequenced green algal genome containing an FBA gene/protein sequence (N0, Fig. 2). Hence, the MRCA of land plants most likely had only a single FBA gene/protein (N1, Fig. 2). This founder gene of the land plant *FBA* gene subfamily was then duplicated early in the lower land plant lineage (N2), resulting in three and seven copies in extant lower land plants *P. patens* and *S. moellendorffii*. In the seed plant lineage, *FBA* genes initially quadrupled (N3) before a more significant expansion occurred after the split of monocots and eudicots in the monocot MRCA (N3). However, reflecting the extent of evolutionary unstable genes described above (Dataset S1), the most dramatic expansion of the *FBA* gene subfamily occurred after the divergence of the various eudicot species. In case of *A. thaliana*, the gene number increased by 20-fold from 9 to 211. This greatly exceeds even the 3-fold increase of the complete FBP superfamily since the divergence of eudicots and monocots 145 million years ago [6].

### Chromosomal organization of *FBA* genes in *A. thaliana*

Tandem gene duplication is known to be one of the the major forces driving the evolution of gene families. Moreover, duplicated genes in a tandem arrangement typically represent more recent duplication events [15]. It therefore seems that tandem duplication may also have played an important role in the above described recent expansion of the *FBA* gene subfamily.

To understand whether *FBA* gene expansion is due to tandem duplications, we used the *A. thaliana* genome as an example, as the gene loci are well mapped for this genome. On the basis of the minimum estimation of linkage disequilibrium in *A. thaliana* [16], we defined two genes as organized in tandem repeats when the distance between them is <10 kb. Vice versa, two genes were defined as not closely linked when they are separated by >10 kb. Based on these criteria, 35% (73 of 207) of the unstable *FBA* genes are organized in tandem repeats. In contrast to this, none of the four stable genes are closely linked to each other (Fig. S4). A similar scenario is observed for the poplar *FBA* genes, for which 44% of the unstable genes are found to be organized in tandem (Dataset S2). This suggests that tandem duplications significantly contributed to the dramatic expansion of *FBA* genes in *A. thaliana* and *P. trichocarpa* since the divergence of eudicot species from their MRCA (Fig. 3). However, for the remaining majority of unstable genes different genetic mechanisms such as segmental or whole genome duplication events must be responsible [17], [18]. Interestingly, 40% of the tandemly repeated *FBA* genes are located in different phylogenetic clades in a species-specific phylogenetic tree for *A. thaliana* (Fig. S4). Again, this illustrates rapid sequence diversification and evolution of this gene family. In support of this hypothesis, we found three proteins with more than one FBA-D (AT1G32140, AT1G26510, AT3G49510), indicating intragenic recombination or duplication due to replication slippage, as described previously for genes in tandem repeats [19].

### Differential selection in *Arabidopsis* spp. *FBA* genes

Having established that unstable and stable genes can be distinguished not only at the level of phylogeny but also at the level of chromosomal organization, we seek to understand whether these differences are also reflected at the level of sequence divergence and signatures of selection.

**Figure 4:**
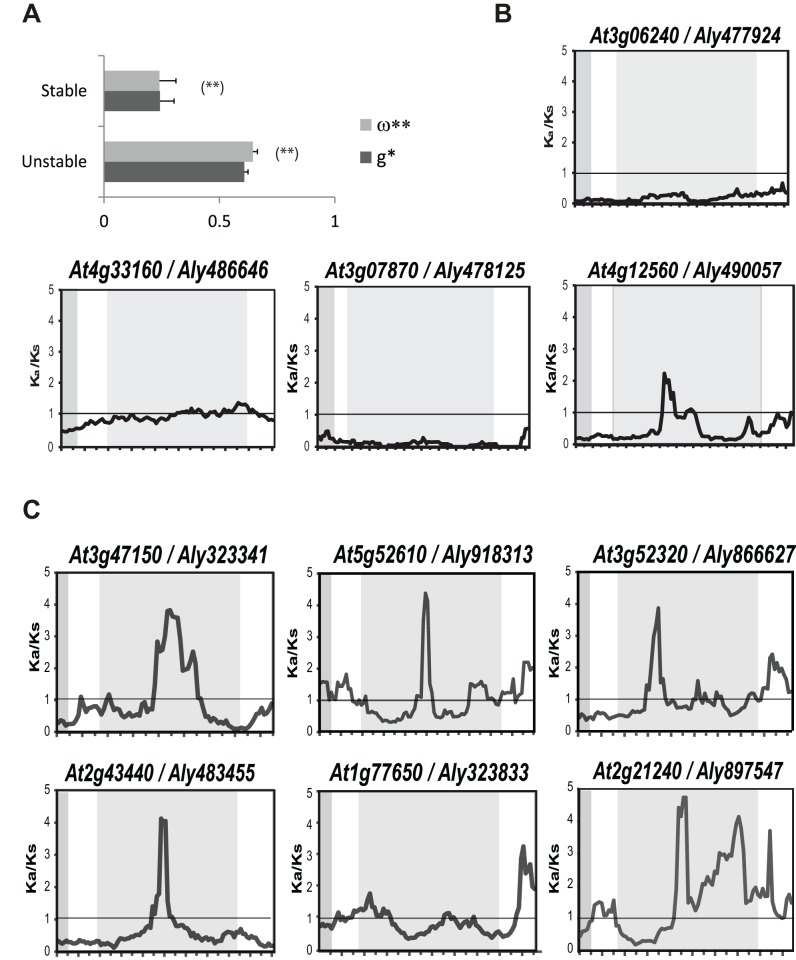
Patterns of selection in *FBA* genes. A, Average *K_a_/K_s_* [ω by Yang [40] and *g* by Comeron [41]] ratios calculated over the complete coding sequence of all stable and unstable genes in *A. thaliana* by comparison to *A. lyrata* orthologs. Error bars represent SE. The difference is significant according to Kruskal-Wallis test (P<0.01). B, Sliding window plots for the four stable genes. C, Sliding window plots for randomly chosen unstable genes. For sliding window analysis, nucleotide sequences of *A. thaliana* (indicated by Arabidopsis Genome Initiative identifier) and orthologous nucleotide sequences of *A. lyrata* (indicated by protein identifier according to the Joint Genome Institute) were used. Window size was 150 bp, and step size was 9 bp. Light gray boxes highlight the position of the F-box domain, and dark gray boxes highlight the FBA-D position.

To investigate whether *FBA* genes with different evolutionary histories (i.e. stable vs. unstable) are subject to different selective pressures, we computed sequence divergence rates of *FBA* genes in form of *K_a_/K_s_* ratios. First, we identified the closest homolog for each *A. thaliana* gene in the genome of the completely sequenced sister species *A. lyrata*. Only those homologs that are clearly orthologs (as evaluated by reciprocal blast and bootstrap support in the phylogenetic tree) are included in the subsequent analyses (Fig. S6). At the level of full-length coding sequence, we found that *K_a_/K_s_* ratios are significantly larger for unstable genes, when compared to stable genes (Fig. 4a; Kruskal-Wallis-test, P<0.01).

To assess whether the same pattern is found for single protein domains, patterns of selection were analyzed by performing a sliding window analysis. In stable genes, as we expected, the F-box domain is highly conserved with *K_a_/K_s_* ratios <<1 (Fig. 4b). With regard to what we had previously learned from the FBK proteins [7], we would have expected the same pattern for the FBA-D. While we largely observed such a pattern for three out of the four stable FBA genes/proteins, the FBA-D of AT4G12560 displays a peak of positively selected amino acid sites at the center of the FBA-D, as observed for most unstable genes. Interestingly, AT4G12560 encodes CPR30/CPR1, a protein that has been reported to be a negative regulator of defense responses in *A. thaliana* [9], [20]. Although the selection signature and the function in biotic stress response suggest adaptive evolution, the conservation of this gene/protein has been reported in another distantly related eudicot species [21].

For unstable *FBA* genes, we find different degrees of selection, reflected in different ranges of *K_a_/K_s_* ratios (Fig. 4C). Nevertheless, a consistent pattern was observed for the conserved F-box domain, which consistently displays *K_a_/K_s_* ratios <1, whereas in the center of the FBA-D there is a tendency to show a *K_a_/K_s_* ratio >>1. We show two exceptions of this overall pattern. One is AT5G52610, which also displays *K_a_/K_s_* >1 in the F-box domain. Other exceptions are two genes, for which the FBA-D displays a *K_a_/K_s_* close to one (AT1G77650 and AT5G51000; Fig. 4C), implying that these domains are not conserved as in the case of stable genes, but seem to evolve neutrally. A possible explanation for this may be pseudogenization. However, we were not able to test this last hypothesis *in silico*, as these genes are not represented on microarrays.

### Expression patterns in *A. thaliana FBA* genes suggest divergence of duplicates at the functional level

**Figure 5:**
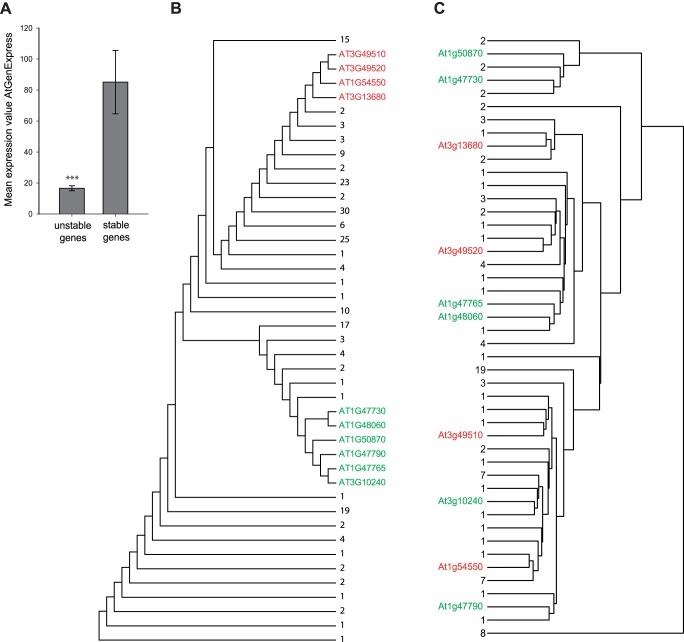
Differential expression profiles of phylogenetically closely related *FBA* genes. A, Mean expression values for unstable and stable *A. thaliana FBA* genes extracted from AtGenExpress_Plus-extended_tissue_series [42]. Error bars represent SE. Statistical significance was assessed using Student´s t-test (***P<0.001). B, NJ tree of 211 *A. thaliana* FBA proteins based on amino acid sequence homology. C, Clustering of 102 *A. thaliana* FBA proteins based on co-expression data from the AtGenExpress_Plus-extended_tissue_series. Discrepancy in the number of FBA genes/proteins between the trees in A and B results from 109 missing *FBA* genes on the ATH1 microarray. Two clades were selected for which all genes had representatives in the ATH1-Chip for illustration purposes (highlighted in green and red font). All other genes were collapsed and labeled according to the number of *FBA* genes within the respective branches.

To further investigate possible events of pseudogenization and functional divergence patterns of stable vs. unstable genes, we next analyzed publicly available global transcriptome data. As mentioned in the last section, we find that the vast majority of *A. thaliana FBA* genes are of an unstable nature, which suggests that many of these genes may either show a very low expression, or have become pseudogenes. In support of this last hypothesis, consultation of published microarray data from the AtGenexpress project ([22]; Dataset S3) shows that unstable *FBA* genes are significantly weaker expressed than stable *FBA* genes (Fig. 5a; Student's t-test, P<0.005). However, most of the unstable *FBA* genes present on the ATH1 microarray can be detected at significant expression levels in several of the 120 tissues assessed. Interestingly, when members of the same phylogenetic clade are clustered according to their expression profiles, they no more group into the same clade (Fig. 5c), which confirms that similar expression patterns are not retained in genes with a common origin. Subfunctionalization of recent copies on the transcriptional level might explain the preservation of numerous *FBA* genes in extant plant genomes following a large scale duplication.

Overall, as already observed in other FBP subfamilies, for instance FBK proteins and tubby-like proteins [7], [23], differential evolution of gene expression appears to be occurring after gene duplication.

## Discussion

FBPs represent one of the largest gene/protein families that spread through the plant kingdom. Hence, a clear picture of their phylogeny and molecular evolution is of special interest for the general understanding of evolutionary histories of multi-domain and/or large protein/gene families in plants. Based on the presence of various C-terminal protein-protein interaction domains, the FBP superfamily can be divided into several subfamilies [4]. As these different C-terminal regions of FBPs are usually not alignable, previous studies relied primarily on the N-terminal F-box domain, which is only ∼50 amino acids long. However, phylogenetic trees based on the F-box alone still clustered proteins with the same C-terminal domains together [4], [6], suggesting that the founder gene of each subfamily has acquired the combination of F-box and C-terminal interaction domain in evolutionary ancient times, and that both domains within a protein co-evolve.

Whenever F-box domains alone are aligned, bootstrap values are rather low. These trees are far from robust. Hence, the phylogenetic signal and information content significantly increases, when alignments include both F-box and the C-terminal domains. We, therefore, provide detailed phylogenetic and evolutionary information on FBPs by analyzing the phylogeny of subfamilies separately. In fact, in *A. thaliana* and other species the largest subfamilies (containing C-terminal leucine-rich repeats, kelch repeats or FBA-Ds) consist of 100+ family members. Thus, reconstructing the molecular phylogeny of these large subfamilies across several plant species represents a challenge by itself.

We have recently analyzed the molecular evolution of FBK proteins in *A. thaliana* [7]. By setting the focus of this study on the largest FBP subfamily, the FBA proteins, we have generated detailed phylogenetic information for an accumulated ∼45% (FBA proteins + FBK proteins) of the complete superfamily (at least in *A. thaliana*). We reason that this will help us understand whether different FBP subfamilies generally share the same evolutionary history or followed rather different paths. Hence, we will focus the discussion of our results on the identification of major evolutionary trends that describe similarities and differences between the two large subfamilies. Our aim was therefore to (i) elucidate evolutionary patterns that may be conserved across the majority of FBPs, and (ii) identify events and characteristics that are specific for certain subfamilies.

### General evolutionary patterns shared by FBP subfamilies

Birth-death evolution and extensive pseudogenization are major forces driving the evolution and expansion of FBPs [2], [6], [14], one of the fastest evolving gene families in the plant kingdom [3]. A model called genomic drift has been invoked to explain a non-directional gain in gene numbers, complementing the gene birth-and-death model [6], [24]. Genomic drift predicts a large intra-species variation in gene numbers of a particular gene family. We did not further explore this possibility, as we did not annotate further *A. thaliana* FBA genes/proteins in populations, the only plant species for which extensive population data are currently available. Nevertheless, although chance might be an important factor, we hypothesize that similar mechanisms keep *FBA* gene copy numbers stable after differential duplication, since closely related species have similar *FBA* gene numbers (Figure 1). We would, therefore, propose the term ‘expansion waves’ to define that not only mode, but also tempo, i.e. timing, of the duplication in a gene families' evolutionary history predicts the evolution of FBPs.

When we seek evidence for rapid evolution on the level of chromosomal organization of *FBA* and *FBK* genes, we observed a general pattern that is shared by both investigated subfamilies. While stable genes are unlinked and evenly scattered over the chromosomes, unstable genes tend to cluster (Fig. S4 and S5). Interestingly, 40% of such tandemly repeated *FBA* genes are located in different phylogenetic clades in an *A. thaliana* specific phylogenetic tree (Fig. S6), illustrating rapid sequence diversification and evolution of this subfamily.

We find similar signatures of rapid evolution on the level of gene expression. In *FBA* and *FBK* gene subfamilies the presumably slowly evolving stable genes are expressed at higher levels and in more tissues than unstable genes (Fig. 5a). Generally, phylogenetically closely related FBA and FBK proteins do not fall into the same groups, when clustered with regard to their gene expression profiles instead of their protein sequence (Fig. 5b, c), demonstrating rapid diversification also on the level of transcriptional regulation. This is a similar pattern of evolution and functional diversification, which has been observed in genes encoding FBP proteins belonging to the S-Locus, the so called *SLF* genes [25], indicating common evolutionary histories of FBP subfamilies. Whether differential expression results in subfunctionalization or pseudogenization of recently duplicated genes is unclear. Possibly, subtle pseudogenization occurs by means of continuous degeneration of the respective *cis*-regulatory regions especially of unstable genes.

Additional common patterns become evident when we consult evolutionary information based on rates of sequence divergence. Not unexpected, *K_a_/K_s_* ratios in evolutionary stable genes are significantly lower across the complete coding sequence than those of evolutionary unstable genes (Fig. 4a). When the F-box and the C-terminal protein-protein interaction domain were evaluated separately, we observed that the F-box domain seems to be under purifying selective pressure (Fig. 4b), possibly to facilitate interaction with the same ASK adapter proteins and therefore incorporation into similar SCF complexes. In contrast, the protein-protein interaction domain shows signatures of positive selection (Fig. 4c), indicating that FBPs might be an important tool for adaptive processes. Our results therefore contradict previous studies on FBPs, where both the F-Box and C-terminal domains appeared to evolve in a correlated manner [5].

The intensity of these selective signatures depends on when in evolutionary time the subfamily expanded significantly. FBK genes/proteins underwent a first wave of expansion early in land plant evolution after the split of green algae and land plants [7], resulting in several hundred million years for the interaction domain to diversify. FBA genes/proteins, however, lack this early wave and – although they are the larger subfamily in several species – expanded later and only in the flowering plant lineage. This is reflected by the grouping of genes/proteins in the phylogenetic trees that include all seven analyzed land plant species showing clustering of orthologs for FBK genes/proteins [7], but clustering of paralogs for *FBA* genes (Fig. 2a). Consequently, the FBA protein subfamily also lacks ancient and superstable genes (Fig. 2b) that were retained in all land plant lineages, as illustrated by the presence of only a single FBA protein sequence in the land plant MRCA (Fig. 3). Accordingly, sliding window analysis of sequence divergence in unstable genes clearly illustrates positive selection acting on the kelch repeat domain of the vast majority of FBK proteins, whereas this pattern is not as consistently present in the FBA-D of FBA proteins.

Together, it seems that FBA and FBK proteins both underwent very similar transitions, including extensive gene family expansion, separation into evolutionary stable genes conserved across lineages and lineage-specific unstable genes, as well as negative and positive selective pressures acting on the F-box or the target recruiting interaction domain, respectively. These trends are likewise shared by both subfamilies on the level of transcription.

### Differences between the evolutionary paths of FBP subfamilies

Besides these global similarities in their evolutionary histories, we observed several, comparably minor, differences between the two subfamilies analyzed. While both subfamilies are also present in non-plant eukaryotes, here only *FBA* genes underwent massive gene family expansions (Table 2); *FBK* genes remain single-copy outside the plant kingdom [7].

**Table 2:** Number of FBA proteins in non-land plant model species.

Species	Number of FBA proteins
*Bacteria*	0
*Chlamydomonas reinhardtii*	0
*Saccharomyces cerevisiae*	0
*Caenorhabditis elegans*	126
*Drosophila melanogaster*	0
*Mus musculus*	21
*Homo sapiens*	10

FBA proteins in non-land plant species were identified by screening the Interpro database from the EMBL-EBI website (http://www.ebi.ac.uk/interpro/) for proteins with the following domains: IPR007397 (F-box associated domain), IPR006527 (F-box associated domain_type1), IPR012885 (F-box associated domain_type2) and IPR013187 (F-box associated domain_type3). Identified proteins were verified for the presence of F-box and FBA domain using Pfam 26.0 [27].

Another obvious difference between FBA and FBK proteins is the homogeneity of their distribution between the species analyzed. While FBK proteins occur in significant numbers across all seven land plant species analyzed [7], the extent of FBA protein subfamily expansion is dramatically different between plant species. In the Brassicaceaes, for example, FBA genes/proteins expanded to >200 subfamily members in each species. Their next closest relative within the Brassicales that was included in this study, *C. papaya*, codes for only 13 FBA proteins, indicating a massive expansion wave after the Brassicaceaes split from the Caricaceaes (Fig. 1). At the lowest end of the spectrum, *S. moellendorffii* contains only three FBA proteins. Taking these differences and the lack of ancient and superstable genes into account, it seems that the vast majority of FBA proteins is not important for basic developmental processes, but may play a role for the adaptation of specific lineages to their respective environments. One example for an FBA protein involved in plant adaptation is the disease resistance regulator CPR30 ([9]; renamed to CPR 1 by Cheng *et* *al.,* [20]). In addition to such unstable genes, the FBK, but not the FBA protein subfamily, also contains a significant number of genes/proteins that are conserved across land plants, and, therefore, possibly play more general roles in land plant development.

## Conclusions

The reported results describe a scenario in which FBP subfamilies generally evolve in a very similar manner. We detected parallels in the general mode of evolution, due to massive duplication and gene birth vs. less incidences of gene death (resulting in stable and unstable genes) and an evolutionary conserved F-box domain compared to a rather divergent C-terminal target recruiting domain. FBP subfamilies tend to expand in waves. Depending on when these dramatic increases in gene number occurred, unstable FBPs display more or less pronounced signatures of positive selection in the target recruiting protein-protein interaction domain, and, thereby, likely confer adaptive potential. While this pattern was rather obvious for FBK proteins [7], this development is possibly currently occurring in FBA proteins, which appear to have expanded much more recently. We hypothesize that it will progressively discriminate the selection signatures of stable and unstable *FBA* genes.

Hence, extensive gene family expansion followed by rapid diversification on the sequence, as well as on the transcriptional level, represent hallmarks of FBP evolution. While unstable FBPs generally follow this pattern, early expansion waves predating higher and lower land plant divergence may additionally generate stable and superstable FBPs that likely play essential roles in general plant development. Given that we observed these evolutionary patterns in the FBA and FBK protein subfamilies, which together comprise ∼45% of the complete FBP superfamily (referring to *A. thaliana*), it is likely that other subfamilies follow similar modes of evolution.

## Materials and Methods

### Identification and verification of FBAs in different land plant genomes

All previously identified FBA protein sequences (*A. thaliana*, *V. vinifera*, *P. trichocarpa*, *O. sativa*, *S. bicolor*, *S. moellendorffii*, *P. patens*) from Schumann *et* *al.* [7] were obtained using a Hidden Markov Model search as implemented in hmmer [26]. FBA protein sequences in the genomes of the 27 additional plant genomes were identified as described in [7]. All potential FBA proteins were then verified for the presence of both F-box and FBA-D using Pfam 26.0 [27]. Sequences that miss one or both of the respective domains were excluded from further analysis. Verified FBA proteins were grouped in clades according to their gene family ID in Plaza 2.0 [28]. Most of these gene families included additional protein sequences. These were also screened for the presence of F-box and FBA-D using Pfam 26.0 resulting in the identification of several new, so far unpublished FBA proteins. All annotated FBA proteins were checked carefully in multiple sequence alignments (described below). Most annotations were correct, except for the protein SB04G026045, which was annotated as containing more than one complete *FBA* gene open reading frame. We therefore split this sequence into two new FBA proteins: SB04G026045 and SB04G026046. All identified FBA proteins that were included in the phylogenetic analysis are listed in Table S1. Table S3 lists the references of all genomes used in this study.

### Identification of FBA proteins in non-plant model species

FBA proteins in non-plant species were identified by screening the Interpro database from the EMBL-EBI website (http://www.ebi.ac.uk/interpro/) for proteins with following domains: IPR007397 (F-box associated domain), IPR006527 (F-box associated domain_type1), IPR012885 (F-box associated domain_type2) and IPR013187 (F-box associated domain_type3). Identified proteins were verified for the presence of F-box and FBA-D using Pfam 26.0 [27].

### Alignment of FBA proteins and phylogenetic reconstruction

FBA proteins from *A. thaliana*, *V. vinifera*, *P. trichocarpa*, *O. sativa*, *S. bicolor*, *S. moellendorffii*, *P. patens,* and *Coccomyxa* sp. C-169 were aligned using MUSCLE [29] with the following parameters: gap extension: −2.9, gap opening: −0.1. The alignment was filtered for informative sites using Gblocks [30], with the option of retaining all gaps sites, as the sequences are largely divergent (overall ID = 20%, except at the domains). The filtered alignment was then used for phylogenetic analysis using maximum likelihood (ML) methods implemented in GARLI (v. 0.95, [13]). We obtained a well supported phylogeny after 2000 runs. A 50% consensus tree was built in MEGA 5 [31]. The accuracy of the ML tree topology was tested by constructing an ML representative tree and subsequent comparison of its topology to trees reconstructed with neighbor joining (NJ) and Bayesian methods. The tree topologies were evaluated with p-SH [32] and 1sKH [33] tests implemented in Treepuzzle [34].

The evolutionary history of 211 *A. thaliana* and 309 *A. lyrata* FBA proteins was inferred using the NJ method [35]. The bootstrap consensus tree inferred from 1000 replicates was used to represent the evolutionary history of the taxa analyzed. The evolutionary distances were computed using the JTT matrix-based method and are in the units of number of amino acid substitutions per site. All positions containing alignment gaps and missing data were eliminated only in pairwise sequence comparisons (Pairwise deletion option). Phylogenetic analyses were conducted in MEGA 5 [31].

### Analysis of selection patterns using sliding windows

Orthologous gene pairs were determined by the protein phylogeny (Fig. S1) and confirmed by reciprocal blast. Pairwise protein alignments were conducted with MAFFT [36] and these alignments were then used as reference for the pairwise alignments using pal2nal [37] needed for DNA coding sequences according to the amino acid alignment. These alignments were fed into DNAsp (v. 5.0, [38]) for *K_a_/K_s_* estimation and sliding window analysis (windowsize = 150 bp, stepsize = 9). The output was fed to an R routine script to extract the information about *K_a_/K_s_* ratios for all pairwise alignments.

### Estimation of the maximum number of gained and lost FBA genes/proteins

To determine the degrees of gene family expansion in the analyzed plant lineages, we divided the phylogeny into ancestral clades (those containing representatives of the lower land plants and angiosperms), recent clades (lower land plant specific, angiosperm specific, monocot specific or eudicot specific), and species-specific clades. It was supposed, that all FBA proteins that are part of an ancestral clade share an MRCA that evolved before the splitting of angiosperms and lower land plants (N1). Subgroups or species that are not represented in these clades are supposed to underlie gene losses. FBA proteins of recent clades are supposed to share an MRCA that evolved before the splitting of monocots and dicots (N2), monocot (N3) or eudicot (N4) species. Only FBA proteins represented in species-specific clades evolved from species-specific ancestors. N0 was added as eukaryotic MRCA on basis of FBA proteins identified in Table 2.

### Correlation analysis of expression data

The expression data of the *A. thaliana FBA* genes were extracted from the AtGenExpress extended tissue series [22], consisting of 120 tissue types. 102 of 211 *A. thaliana FBA* genes were represented on the ATH1 microarray. The extracted data were log2 transformed to achieve normal distribution. For the hierarchical cluster analysis of the expression data, we used the R package pvclust [39]. In pvclust, the dendrograms were created with the UPGMA method (average linkage) as cluster distance function. To measure the similarity between genes, we calculated euclidean distances that take also differences in the expression level into account. In parallel, Pearson correlation was used to reveal genes with similar response.

## Supporting Information

Figure S1 **Representative alignment of 24 randomly chosen FBA proteins used for tree reconstruction.** The alignment was obtained with MUSCLE. It was used for phylogenetic reconstruction according to NJ and Bayesian methods (Fig. S2) to confirm the robustness of the ML tree used in the main analysis (Fig. 2a). The amino acids composing the F-Box domain are marked with a dashed line above the alignment (positions 1–40), those composing the FBA domain are enclosed in a box (pos. 290–520).
(EPS)

Figure S2 **Representative trees generated from 24 representative FBA protein sequences.** The original alignment was obtained with MUSCLE. Tree reconstruction was done using the NJ (A), Bayesian (B), and ML method (C). 1000 bootstrap replicates were used for the NJ and ML methods. For the Bayesian tree 50,000 generations were run to obtain a standard deviation probability <0.05.
(EPS)

Figure S3 **Linear phylogenetic tree of** ***A. thaliana, V. vinifera, P. trichocarpa, O. sativa, S. bicolor, S. moellendorffii and*** ** ***P. patens*** **FBA proteins.** Multiple alignments of the full-lenght FBA protein sequences were performed using MUSCLE. The phylogenetic tree was generated with ML methods implemented in GARLI (likelihood = −91644.250131 after 2000 bootstrap runs). The tree was rooted with an FBA of *Coccomyxa* sp. C−169. The color code corresponds to the different species. Numbers at branches indicate bootstrap values >40. The tree was divided in 26 clades according to the bootstrap support and gene family information from Plaza 2.0 [28], respectively. With exception of clades 15 and 26, FBA proteins are considered to cluster together if they have a bootstrap support >40. FBA proteins of clade 15 and 26 were clustered according to Plaza 2.0 information. The FBAs of clade 15 are members of the Plaza gene family HOM000168 and the FBA proteins of clade 26 are members of the Plaza gene family HOM000207. The clades were categorized in (i) unstable: lineage-specific clades, (ii) stable: clades including orthologs in at least two species, and (iii) singleton: FBA proteins that do not cluster together with other FBA proteins.
(EPS)

Figure S4 **Chromosomal organization of** ***FBA*** **genes in** ***A. thaliana*** *.* Approximate positions of *FBA* genes are displayed on the respective chromosome. Black letters indicate unstable *FBA* genes, red letters stable and green letters singleton *FBA* genes. Classification of *FBA* genes in unstable, stable and singleton is according to the cluster analysis presented in Fig. S2.
(EPS)

Figure S5 **Chromosomal organization of** ***FBA*** **gene homology groups in** ***A.*** ** ***thaliana*** *.* Genes in homology groups are not in tandem repeats, but are localized in different genomic locations. Positions of *FBA* genes are displayed on the respective chromosome, black bars indicate unstable *FBA* genes, red bars indicate stable *FBA* genes, yellow bars indicate singleton *FBA* genes. Only genes from the largest homology group HOM000207 (Dataset S1) and the smallest homology group HOM007652 are displayed as red and blue line(s) in the center of the circle plot, respectively.
(EPS)

Figure S6 **50% consensus tree of 211** ***A. thaliana*** **and 309** ***A. lyrata*** **FBA proteins.** The tree was constructed in MEGA 5 [31] using the NJ algorithm [35]. 1000 bootstrap replicates were used, the tree indicates the topology and does not display branch lengths for better visualization. Enclosed in brackets are 132 orthologous duplicated genes confirmed by the phylogenetic tree and/or best reciprocal blast hits.
(EPS)

Table S1 Identifiers of F-box proteins with F-box associated domains in *A. thaliana*, *P. trichocarpa*, *V. vinifera*, *O. sativa*, *S. bicolor*, *S. moellendorffii* and *P. patens*.
(DOC)

Table S2 Comparison of three tree topologies obtained with three different methods (NJ  =  Neighbor-Joining, ML  =  Maximum Likelihood and Bayesian) using Shimodaira-Hasegawa (p-SH, [32]) and one sided Kishino-Hasegawa (1sKH, [33]) tests, implemented in Treepuzzle [34]. Additionally, a Chi-square test was performed to compare the likelihoods (l) of the three trees to the best tree (in this case the Bayesian tree). The NJ and ML trees have a better likelihood, but are not significantly better than the Bayesian tree.
(DOC)

Table S3 References for the genome sequences used to mine for *FBA* genes.
(DOC)

Dataset S1 (XLS)

Dataset S2 (XLS)

Dataset S3 (XLS)
