# Supplementary material for: Plant F-Box Protein Evolution Is Determined by Lineage-Specific Timing of Major Gene Family Expansion Waves
Source: PLoS One. 2013 Jul 19;8(7):e68672. doi: 10.1371/journal.pone.0068672 (PMC3719486; doi:10.1371/journal.pone.0068672)
Supplement: Table S1 — Identifiers of F-box proteins with F-box associated domains in A. thaliana, P. trichocarpa, V. vinifera, O. sativa, S. bicolor, S. moellendorffii and P. patens. (DOC) [file pone.0068672.s007.doc]

| **Table S1.** Identifiers of F-box proteins with F-box associated domains in *A. thaliana*, *P. trichocarpa, V. vinifera*, *O. sativa*, *S. bicolor*, *S. moellendorffii* and *P. patens.* | | | | | | |
| --- | --- | --- | --- | --- | --- | --- |
| *A. thaliana* | *P. trichocarpa* | *V. vinifera* | *O. sativa* | *S. bicolor* | *S. moellendorffii* | *P. patens* |
| AT1G09650 | PT00G00150 | VV00G26390 | OS02G10600 | SB01G003460 | SM00020G00070 | PP00004G01380 |
| AT1G11270 | PT00G20680 | VV05G01300 | OS02G33840 | SB01G013220 | SM00036G02730 | PP00052G00340 |
| AT1G11620 | PT01G00950 | VV12G12100 | OS02G35560 | SB01G013240 | SM00101G00940 | PP00066G00750 |
| AT1G11810 | PT01G00970 | VV15G05810 | OS03G25640 | SB01G018980 |  | PP00129G00630 |
| AT1G12170 | PT01G07090 | VV17G04830 | OS03G46690 | SB01G031390 |  | PP00153G00370 |
| AT1G12190 | PT01G22390 | VV18G05960 | OS04G11450 | SB01G034970 |  | PP00198G00100 |
| AT1G12870 | PT01G31810 |  | OS04G11660 | SB01G037880 |  | PP00217G00290 |
| AT1G13200 | PT01G31820 |  | OS04G11790 | SB02G001040 |  |  |
| AT1G14315 | PT01G39770 |  | OS04G50200 | SB02G005020 |  |  |
| AT1G19160 | PT01G45310 |  | OS05G02570 | SB02G005060 |  |  |
| AT1G24881 | PT02G11760 |  | OS05G08010 | SB02G005100 |  |  |
| AT1G25141 | PT02G21670 |  | OS05G08350 | SB02G005870 |  |  |
| AT1G26510 | PT03G05100 |  | OS05G08440 | SB02G005880 |  |  |
| AT1G27490 | PT04G00070 |  | OS05G08460 | SB02G005890 |  |  |
| AT1G30780 | PT05G26460 |  | OS06G07380 | SB02G008113 |  |  |
| AT1G30790 | PT06G01110 |  | OS06G07460 | SB02G023440 |  |  |
| AT1G30920 | PT06G01180 |  | OS07G08570 | SB02G027685 |  |  |
| AT1G30930 | PT06G17060 |  | OS07G09710 | SB02G027700 |  |  |
| AT1G31000 | PT07G07250 |  | OS07G09814 | SB03G044080 |  |  |
| AT1G31080 | PT08G00550 |  | OS07G09870 | SB04G000800 |  |  |
| AT1G31090 | PT08G03600 |  | OS07G13870 | SB04G003010 |  |  |
| AT1G32140 | PT08G14110 |  | OS07G13890 | SB04G006790 |  |  |
| AT1G32420 | PT08G20240 |  | OS07G16420 | SB04G025085 |  |  |
| AT1G32430 | PT08G21000 |  | OS07G16800 | SB04G026045 |  |  |
| AT1G32660 | PT08G22150 |  | OS07G35050 | SB05G020470 |  |  |
| AT1G33010 | PT08G22370 |  | OS07G35060 | SB05G025730 |  |  |
| AT1G33020 | PT10G14750 |  | OS08G10340 | SB05G025750 |  |  |
| AT1G33530 | PT10G20310 |  | OS08G36960 | SB06G027040 |  |  |
| AT1G46840 | PT11G03590 |  | OS08G38490 | SB08G003880 |  |  |
| AT1G46984 | PT11G11420 |  | OS09G20650 | SB08G015610 |  |  |
| *A. thaliana* | *P. trichocarpa* | *V. vinifera* | *O. sativa* | *S. bicolor* | *S. moellendorffii* | *P. patens* |
| AT1G47340 | PT11G13270 |  | OS09G27570 | SB08G021860 |  |  |
| AT1G47390 | PT12G01970 |  | OS09G30180 | SB08G021900 |  |  |
| AT1G47730 | PT12G02000 |  | OS09G34200 | SB08G022710 |  |  |
| AT1G47765 | PT13G04830 |  | OS10G04850 | SB09G027720 |  |  |
| AT1G47790 | PT13G08850 |  | OS10G25210 | SB09G030410 |  |  |
| AT1G47800 | PT13G09490 |  | OS10G25660 | SB10G010900 |  |  |
| AT1G47810 | PT14G01460 |  | OS12G03440 | SB10G024600 |  |  |
| AT1G48060 | PT14G15870 |  | OS12G06740 |  |  |  |
| AT1G50870 | PT14G19140 |  | OS12G30920 |  |  |  |
| AT1G50880 | PT15G01390 |  |  |  |  |  |
| AT1G51290 | PT15G01430 |  |  |  |  |  |
| AT1G51320 | PT16G01220 |  |  |  |  |  |
| AT1G52490 | PT17G08290 |  |  |  |  |  |
| AT1G53370 | PT17G08370 |  |  |  |  |  |
| AT1G53550 | PT17G10020 |  |  |  |  |  |
| AT1G53790 | 548689 |  |  |  |  |  |
| AT1G54550 | 587549 |  |  |  |  |  |
| AT1G55070 |  |  |  |  |  |  |
| AT1G58090 |  |  |  |  |  |  |
| AT1G59680 |  |  |  |  |  |  |
| AT1G60370 |  |  |  |  |  |  |
| AT1G61060 |  |  |  |  |  |  |
| AT1G62270 |  |  |  |  |  |  |
| AT1G65990 |  |  |  |  |  |  |
| AT1G66490 |  |  |  |  |  |  |
| AT1G67130 |  |  |  |  |  |  |
| AT1G67450 |  |  |  |  |  |  |
| AT1G67455 |  |  |  |  |  |  |
| AT1G70380 |  |  |  |  |  |  |
| AT1G70390 |  |  |  |  |  |  |

| *A. thaliana* | *P. trichocarpa* | *V. vinifera* | *O. sativa* | *S. bicolor* | *S. moellendorffii* | *P. patens* |
| --- | --- | --- | --- | --- | --- | --- |
| AT1G70960 |  |  |  |  |  |  |
| AT1G70970 |  |  |  |  |  |  |
| AT1G71320 |  |  |  |  |  |  |
| AT1G76830 |  |  |  |  |  |  |
| AT1G77650 |  |  |  |  |  |  |
| AT2G02030 |  |  |  |  |  |  |
| AT2G02660 |  |  |  |  |  |  |
| AT2G02890 |  |  |  |  |  |  |
| AT2G04920 |  |  |  |  |  |  |
| AT2G07140 |  |  |  |  |  |  |
| AT2G14710 |  |  |  |  |  |  |
| AT2G15640 |  |  |  |  |  |  |
| AT2G16220 |  |  |  |  |  |  |
| AT2G16450 |  |  |  |  |  |  |
| AT2G16810 |  |  |  |  |  |  |
| AT2G17310 |  |  |  |  |  |  |
| AT2G17830 |  |  |  |  |  |  |
| AT2G18780 |  |  |  |  |  |  |
| AT2G19630 |  |  |  |  |  |  |
| AT2G23160 |  |  |  |  |  |  |
| AT2G24510 |  |  |  |  |  |  |
| AT2G27520 |  |  |  |  |  |  |
| AT2G31470 |  |  |  |  |  |  |
| AT2G33655 |  |  |  |  |  |  |
| AT2G34280 |  |  |  |  |  |  |
| AT2G38590 |  |  |  |  |  |  |
| AT2G40910 |  |  |  |  |  |  |
| AT2G40920 |  |  |  |  |  |  |
| AT2G40925 |  |  |  |  |  |  |
| AT2G43260 |  |  |  |  |  |  |

| *A. thaliana* | *P. trichocarpa* | *V. vinifera* | *O. sativa* | *S. bicolor* | *S. moellendorffii* | *P. patens* |
| --- | --- | --- | --- | --- | --- | --- |
| AT2G43440 |  |  |  |  |  |  |
| AT2G43445 |  |  |  |  |  |  |
| AT3G04660 |  |  |  |  |  |  |
| AT3G06240 |  |  |  |  |  |  |
| AT3G07870 |  |  |  |  |  |  |
| AT3G08750 |  |  |  |  |  |  |
| AT3G10240 |  |  |  |  |  |  |
| AT3G10430 |  |  |  |  |  |  |
| AT3G10790 |  |  |  |  |  |  |
| AT3G13680 |  |  |  |  |  |  |
| AT3G13820 |  |  |  |  |  |  |
| AT3G13830 |  |  |  |  |  |  |
| AT3G16210 |  |  |  |  |  |  |
| AT3G16555 |  |  |  |  |  |  |
| AT3G16580 |  |  |  |  |  |  |
| AT3G16590 |  |  |  |  |  |  |
| AT3G16740 |  |  |  |  |  |  |
| AT3G16820 |  |  |  |  |  |  |
| AT3G16880 |  |  |  |  |  |  |
| AT3G17265 |  |  |  |  |  |  |
| AT3G17280 |  |  |  |  |  |  |
| AT3G17320 |  |  |  |  |  |  |
| AT3G17480 |  |  |  |  |  |  |
| AT3G17490 |  |  |  |  |  |  |
| AT3G17500 |  |  |  |  |  |  |
| AT3G17530 |  |  |  |  |  |  |
| AT3G17540 |  |  |  |  |  |  |
| AT3G17560 |  |  |  |  |  |  |
| AT3G17570 |  |  |  |  |  |  |
| AT3G17620 |  |  |  |  |  |  |

| *A. thaliana* | *P. trichocarpa* | *V. vinifera* | *O. sativa* | *S. bicolor* | *S. moellendorffii* | *P. patens* |
| --- | --- | --- | --- | --- | --- | --- |
| AT3G17710 |  |  |  |  |  |  |
| AT3G18320 |  |  |  |  |  |  |
| AT3G18330 |  |  |  |  |  |  |
| AT3G18340 |  |  |  |  |  |  |
| AT3G18910 |  |  |  |  |  |  |
| AT3G18980 |  |  |  |  |  |  |
| AT3G19410 |  |  |  |  |  |  |
| AT3G19470 |  |  |  |  |  |  |
| AT3G19560 |  |  |  |  |  |  |
| AT3G19880 |  |  |  |  |  |  |
| AT3G19890 |  |  |  |  |  |  |
| AT3G20030 |  |  |  |  |  |  |
| AT3G20690 |  |  |  |  |  |  |
| AT3G20710 |  |  |  |  |  |  |
| AT3G21120 |  |  |  |  |  |  |
| AT3G21130 |  |  |  |  |  |  |
| AT3G21170 |  |  |  |  |  |  |
| AT3G21410 |  |  |  |  |  |  |
| AT3G22350 |  |  |  |  |  |  |
| AT3G22421 |  |  |  |  |  |  |
| AT3G22650 |  |  |  |  |  |  |
| AT3G22700 |  |  |  |  |  |  |
| AT3G22710 |  |  |  |  |  |  |
| AT3G22720 |  |  |  |  |  |  |
| AT3G22730 |  |  |  |  |  |  |
| AT3G22870 |  |  |  |  |  |  |
| AT3G22940 |  |  |  |  |  |  |
| AT3G23260 |  |  |  |  |  |  |
| AT3G23420 |  |  |  |  |  |  |
| AT3G23880 |  |  |  |  |  |  |

| *A. thaliana* | *P. trichocarpa* | *V. vinifera* | *O. sativa* | *S. bicolor* | *S. moellendorffii* | *P. patens* |
| --- | --- | --- | --- | --- | --- | --- |
| AT3G23960 |  |  |  |  |  |  |
| AT3G24580 |  |  |  |  |  |  |
| AT3G24700 |  |  |  |  |  |  |
| AT3G25460 |  |  |  |  |  |  |
| AT3G44120 |  |  |  |  |  |  |
| AT3G44130 |  |  |  |  |  |  |
| AT3G47020 |  |  |  |  |  |  |
| AT3G47030 |  |  |  |  |  |  |
| AT3G47150 |  |  |  |  |  |  |
| AT3G49450 |  |  |  |  |  |  |
| AT3G49510 |  |  |  |  |  |  |
| AT3G49520 |  |  |  |  |  |  |
| AT3G49980 |  |  |  |  |  |  |
| AT3G51171 |  |  |  |  |  |  |
| AT3G52320 |  |  |  |  |  |  |
| AT3G57580 |  |  |  |  |  |  |
| AT3G57590 |  |  |  |  |  |  |
| AT3G59610 |  |  |  |  |  |  |
| AT3G61340 |  |  |  |  |  |  |
| AT4G04690 |  |  |  |  |  |  |
| AT4G05080 |  |  |  |  |  |  |
| AT4G09190 |  |  |  |  |  |  |
| AT4G09870 |  |  |  |  |  |  |
| AT4G10190 |  |  |  |  |  |  |
| AT4G10740 |  |  |  |  |  |  |
| AT4G11590 |  |  |  |  |  |  |
| AT4G12560 |  |  |  |  |  |  |
| AT4G17200 |  |  |  |  |  |  |
| AT4G17780 |  |  |  |  |  |  |
| AT4G19930 |  |  |  |  |  |  |

| *A. thaliana* | *P. trichocarpa* | *V. vinifera* | *O. sativa* | *S. bicolor* | *S. moellendorffii* | *P. patens* |
| --- | --- | --- | --- | --- | --- | --- |
| AT4G19940 |  |  |  |  |  |  |
| AT4G21240 |  |  |  |  |  |  |
| AT4G29970 |  |  |  |  |  |  |
| AT4G33160 |  |  |  |  |  |  |
| AT4G33290 |  |  |  |  |  |  |
| AT4G38870 |  |  |  |  |  |  |
| AT5G07610 |  |  |  |  |  |  |
| AT5G10340 |  |  |  |  |  |  |
| AT5G15660 |  |  |  |  |  |  |
| AT5G15670 |  |  |  |  |  |  |
| AT5G18160 |  |  |  |  |  |  |
| AT5G22791 |  |  |  |  |  |  |
| AT5G36200 |  |  |  |  |  |  |
| AT5G36730 |  |  |  |  |  |  |
| AT5G37040 |  |  |  |  |  |  |
| AT5G38810 |  |  |  |  |  |  |
| AT5G41490 |  |  |  |  |  |  |
| AT5G41500 |  |  |  |  |  |  |
| AT5G41510 |  |  |  |  |  |  |
| AT5G42430 |  |  |  |  |  |  |
| AT5G42460 |  |  |  |  |  |  |
| AT5G47300 |  |  |  |  |  |  |
| AT5G50220 |  |  |  |  |  |  |
| AT5G51000 |  |  |  |  |  |  |
| AT5G52610 |  |  |  |  |  |  |
| AT5G52620 |  |  |  |  |  |  |
| AT5G60560 |  |  |  |  |  |  |
| AT5G62060 |  |  |  |  |  |  |
| AT5G62510 |  |  |  |  |  |  |
| AT5G62660 |  |  |  |  |  |  |
| AT5G65850 |  |  |  |  |  |  |
