# Supplementary material for: Plant F-Box Protein Evolution Is Determined by Lineage-Specific Timing of Major Gene Family Expansion Waves
Source: PLoS One. 2013 Jul 19;8(7):e68672. doi: 10.1371/journal.pone.0068672 (PMC3719486; doi:10.1371/journal.pone.0068672)
Supplement: Table S2 — Comparison of three tree topologies obtained with three different methods (NJ = Neighbor-Joining, ML = Maximum Likelihood and Bayesian) using Shimodaira-Hasegawa (p-SH, [32]) and one sided Kishino-Hasegawa (1sKH, [33]) tests, implemented in Treepuzzle [34]. Additionally, a Chi-square test was performed to compare the likelihoods (l) of the three trees to the best tree (in this case the Bayesian tree). The NJ and ML trees have a better likelihood, but are not significantly better than the Bayesian tree. (DOC) [file pone.0068672.s008.doc]

| **Table S2.** Comparison of tree topologies obtained with three different methods (NJ= Neighbor Joining, ML = Maximum Likelihood and Bayesian), using Shimodaira-Hasegawa (p-SH, Shimodaira-Hasegawa, 1999) and one sided Kishino-Hasegawa (1sKH, Kishino-Hasegawa, 1989) tests, implemented in Treepuzzle (Schmidt & von Haeseler, 2007). Additionally, a Chi-square test was performed to compare the likelihoods (l) of the three trees to the best tree (in this case the Bayesian tree). NJ and ML trees have a lower likelihood, but are not significantly better than the Bayesian tree. | | | | | | | | |
| --- | --- | --- | --- | --- | --- | --- | --- | --- |
| Tree | l | Δ l | S.E. | p1-skh | p-SH | 2Δl | Chi-square | p-value |
| NJ | -21962 | 43.84 | 16.12 | 0.004 | 0.009 | 87.68 | 0.0877 | 0.7671 |
| ML | -22030.09 | 111.93 | 20.32 | 0 | 0 | 223.86 | 0.5716 | 0.4496 |
| Bayesian | -21918.16 | 0 | best tree | 0 | 1 | 0 | 0 | - |
