# Supplementary material for: Plant F-Box Protein Evolution Is Determined by Lineage-Specific Timing of Major Gene Family Expansion Waves
Source: PLoS One. 2013 Jul 19;8(7):e68672. doi: 10.1371/journal.pone.0068672 (PMC3719486; doi:10.1371/journal.pone.0068672)
Supplement: Table S3 — References for the genome sequences used to mine for FBA genes. (DOC) [file pone.0068672.s009.doc]

**Table S3.** References for the genome sequences used to mine for FBA genes.

| *Species name* | Reference for genome sequence data |
| --- | --- |
| *Arabidopsis lyrata* | Hu T et al. (2011). The *Arabidopsis lyrata* genome sequence and the basis of rapid genome size change. Nature Genetics 43: 476–481. |
| *Arabidopsis thaliana* | The Arabidopsis Genome Initiative (2000). Analysis of the genome sequence of the flowering plant *Arabidopsis thaliana*. Nature, 408: 796-815. |
| *Brachipodium distachyon* | Vogel J et al. (2010). Genome sequencing and analysis of the model grass *Brachypodium distachyon*. Nature, 463: 763-768. |
| *Brassica rapa* | Wang X et al. (2011). The genome of the mesopolyploid crop species *Brassica rapa*. Nature Genetics 43: 1035-1039. |
| *Carica papaya* | Ming R et al. (2008). The draft genome of the transgenic tropical fruit tree papaya (*Carica papaya* Linnaeus). Nature, 452: 991-996. |
| *Citrus clementina* | Haploid Clementine Genome, International Citrus Genome Consortium, 2011, http://int-citrusgenomics.org/, http://www.phytozome.net/clementine |
| *Citrus sinensis* | Sweet Orange Genome Project 2010, International Citrus Genome Consortium, http::// www.phytozome.net/orange |
| *Cucumis sativus* | Huang S et al. (2009). The genome of the cucumber, *Cucumis sativus* L. Nature Genetics 41: 1275-1281. |
| *Fragaria vesca* | Shulaev V et al. (2012). The genome of woodland strawberry (*Fragaria vesca*). Nature Genetics 43: 109-116. |
| *Glycine max* | Schmutz J et al. (2010). Genome sequence of the palaeopolyploid soybean. Nature 463: 178-183. |
| *Gossypium raimondii* | Wang K et al. (2012). The draft genome of a diploid cotton *Gossypium raimondii*. Nature Genetics 44: 1098–1103. |
| *Linum usitatissimum* | Wang Z et al. (2012). The genome of flax (*Linum usitatissimum*) assembled de novo from short shotgun sequence reads. The Plant Journal 72: 461-473. |
| *Malus domestica* | Velasco R et al. (2010). The genome of the domesticated apple (*Malus domestica* Borkh.). Nature Genetics 42: 833–839. |
| *Manihot esculenta* | Prochnik S et al. (2012). The Cassava Genome: Current Progress, Future Directions. Tropical Plant Biology 55: 88-94. |
| *Medicago truncatula* | Young ND et al. (2011). The Medicago genome provides insight into the evolution of rhizobial symbioses. Nature 480: 520-524. |
| *Micromonas pusilla CCMP1545* | Worden et al. (2009). Green Evolution and Dynamic Adaptations Revealed by Genomes of the Marine Picoeukaryotes *Micromonas*. Science 324: 268-272. |
| *Micromonas pusilla RCC299* | Worden et al. (2009). Green Evolution and Dynamic Adaptations Revealed by Genomes of the Marine Picoeukaryotes *Micromonas*. Science 324: 268-272. |
| *Oryza sativa* | Goff S et al. (2002). A Draft Sequence of the Rice Genome (*Oryza sativa* L. ssp. japonica). Science 296: 92-100. |
| *Ostreococcus lucmarinus* | Palenik B et al. (2007). The tiny eukaryote *Ostreococcus* provides genomic insights into the paradox of plankton speciation. Proceedings of the Natl Acad Sci U S A 104: 7705-7710. |
| *Physcomitrella patens* | Rensing SA et al. (2008). The *Physcomitrella* genome reveals evolutionary insights into the conquest of land by plants. Science 319 :64-69. |
| *Populus trichocarpa* | Merchant S et al. (2007). The *Chlamydomonas* Genome Reveals the Evolution of Key Animal and Plant Functions. Science 318: 245-250. |
| *Prunus persica* | Jung S et al. (2012). Whole genome comparisons of *Fragaria*, *Prunus* and *Malus* reveal different modes of evolution between Rosaceous subfamilies. BMC Genomics 13: 129. |
| *Ricinus communis* | Chan AP et al. (2010). Draft genome sequence of the oilseed species *Ricinus communis*. Nature Biotechnology 28: 951–956. |
| *Selaginella moellendorffii* | Banks et al. (2011). The *Selaginella* Genome Identifies Genetic Changes Associated with the Evolution of Vascular Plants. Science 332:960-963. |
| *Setaria italica* | Zheng G et al. (2012). Genome sequence of foxtail millet (*Setaria italica*) provides insights into grass evolution and biofuel potential. Nature Biotechnology 30: 549–554. Bennetzen J et al. (2012). Reference genome sequence of the model plant *Setaria*. Nature Biotechnology 30: 555–561. |
| *Solanum lycopersicum* | The Tomato Genome Consortium (2012). The tomato genome sequence provides insights into fleshy fruit evolution. Nature 485: 635–641. |
| *Solanum tuberosum* | The Potato Genome Sequencing Consortium (2011). Genome sequence and analysis of the tuber crop potato. Nature 475: 189–195. |
| *Sorghum bicolor* | Paterson A et al. (2009). The *Sorghum bicolor* genome and the diversification of grasses. Nature 457: 551-556. |
| *Theobroma cacao* | Argout X et al. (2011). The genome of *Theobroma cacao*. Nature Genetics 43: 101-108. |
| *Vitis vinifera* | Jaillon O et al. (2007). The grapevine genome sequence suggests ancestral hexaploidization in major angiosperm phyla. Nature 449, 463-467. |
| *Zea mays* | Schnable P et al. (2009). The B73 Maize Genome: Complexity, Diversity, and Dynamics. Science 326: 1112-1115. |
